# The Association between Charlson Comorbidity Index and the Medical Care Cost of Cancer: A Retrospective Study

**DOI:** 10.1155/2015/259341

**Published:** 2015-08-04

**Authors:** Seok-Jun Yoon, Eun-Jung Kim, Hyun-Ju Seo, In-Hwan Oh

**Affiliations:** ^1^Department of Preventive Medicine, College of Medicine, Korea University, Anam-dong, Seongbuk-gu, Seoul 136-705, Republic of Korea; ^2^Department of Economics, Economics Research Institute, Korea University, Anam-dong, Seongbuk-gu, Seoul 136-705, Republic of Korea; ^3^Department of Nursing, College of Medicine, Chosun University, Dong-gu, Gwangju 61452, Republic of Korea; ^4^Department of Preventive Medicine, College of Medicine, Kyung Hee University, Dongdaemun-gu, Seoul 130-701, Republic of Korea

## Abstract

*Background*. This study compared comorbidity-related medical care cost associated with different types of cancer, by examining breast (*N* = 287), colon (*N* = 272), stomach (*N* = 614), and lung (*N* = 391) cancer patients undergoing surgery. *Methods*. Using medical benefits claims data, we calculated Charlson Comorbidity Index (CCI) and total medical cost. The effect of comorbidity on the medical care cost was investigated using multiple regression and logistic regression models and controlling for demographic characteristics and cancer stage. *Results*. The treatment costs incurred by stomach and colon cancer patients were 1.05- and 1.01-fold higher, respectively, in patients with higher CCI determined. For breast cancer, the highest costs were seen in those with chronic obstructive pulmonary disease (COPD), but the increase in cost reduced as CCI increased. Colon cancer patients with diabetes mellitus and a CCI = 1 score had the highest medical costs. The lowest medical costs were incurred by lung cancer patients with COPD and a CCI = 2 score. *Conclusion*. The comorbidities had a major impact on the use of medical resources, with chronic comorbidities incurring the highest medical costs. The results indicate that comorbidities affect cancer outcomes and that they must be considered strategies mitigating cancer's economic and social impact.

## 1. Introduction

The burden of cancer has increased annually, and the disease has been the leading cause of death for 21 years, as reported by Statistics Korea. In 2008, the number of cancer deaths was 3-4 times higher (139.5 per 100,000 cases) than mortality associated with cerebrovascular and cardiovascular diseases (56.5 and 43.4 per 100,000 cases, resp.) [1]. Regarding the burden of diseases in Korea, total cancers are the major cause of burden among chronic diseases [2]. Since Korea's elderly population is growing at a faster rate than other members of the Organization for Economic Co-Operation and Development (OECD), the burden of cancer is expected to rise, and so measures to manage the burden of disease must be implemented. Cancer incidence tends to increase among older populations, as does the risk of comorbidity; elderly cancer patients (aged over 70 years) have, on average, over three comorbidities [3, 4]. These associated diseases have a negative effect on health outcomes by extending hospital length of stay, increasing postoperative complications, and increasing mortality rate [5–7]. It is therefore important to evaluate the burden of cancer while considering comorbidities and to implement measures to manage the associated social and economic costs.

Severity and fatality of a disease such as cancer can be examined by investigating comorbidity. Janssen-Heijnen et al. reported that, for certain cancer types (e.g., lung cancer) whose operation risk necessitates treatment with alternative therapies, the treatment method is determined according to the existence and the degree of comorbidity, to ensure that it did not affect the treatment outcome [8]. However, for cancer types requiring surgical treatment (e.g., colon and breast cancers), the existence of comorbidity does not influence treatment options, and various comorbidities showed an improved survival rate [8]. Variations in the fatality rate of certain cancer types affect overall cancer mortality patterns. For example, cancer types generally accompanied by a good prognosis are more likely to occur with comorbidities, and 24.0% of deaths occurring five years after diagnosis have been caused by comorbidities; therefore, managing and treating comorbidities should be considered as an important way to improve the health outcomes of cancer patients [9–11].

So, this study examined the medical care cost of patients with various types of cancer based on the number and type of comorbidities. Using medical cost as a measure of the economic burden, we also examined the effect of chronic or acute comorbidities on the medical care cost of cancer patients.

## 2. Methods

### 2.1. Patients

This retrospective, noncontrolled, and nonrandomized study examined patients undergoing surgery (excluding endoscopic mucosal resection) for breast (*N* = 287), colon (*N* = 272), stomach (*N* = 614), and lung (*N* = 391) cancers. Surgery took place at the Korea University Anam Hospital from January 2005 to December 2007 (breast and stomach cancers) and January 2005 to August 2008 (colon cancer) or at the Korea National Cancer Center from January 2000 to December 2004 (lung cancer). Patients with rectal cancer were not included in the colon cancer group. The leading types of cancer in Korea are stomach, lung, liver, colon, and breast cancers; liver cancer was excluded from this study because surgical treatments that do not include organ transplantation are rarely performed, due to the availability of other liver cancer therapies, and so recruiting a sufficient number of subjects was not possible.

### 2.2. Comorbidity Indexes

The Charlson Comorbidity Index (CCI) was used to estimate health outcomes and was measured using the International Classification of Diseases- (ICD-) 10 code [12]. To investigate comorbidities using claims data, we used comorbidities lasting for two years prior to the cancer diagnosis as reported by the claims data of outpatients and inpatients in the Electronic Data Interchange (EDI) of the Health Insurance Review and Assessment Service (HIRA), as well as the ICD-10 codes of the associated diseases [13–15]. Any disease that had been diagnosed two or more times in the benefits claims data for the study period was considered a comorbidity by applying the algorithm of Klabunde et al. to increase diagnostic accuracy [16–18].

### 2.3. Measurement of Medical Care Cost

Total medical care cost was defined as all medical benefits for surgery-related hospitalization and outpatient cost like chemotherapy and follow-up care cost based on the medical care claims found in the HIRA EDI. Since the study period covered 2000–2010, all spending costs were adjusted based on the 2010 price index.

### 2.4. Statistical Analysis

Multiple regression models were used to investigate the effect of comorbidity on the medical care cost. Total medical care cost was biased toward the right side, and so it was converted into logarithm. This study controlled for demographic characteristics and cancer stage to estimate the influence of CCI on health outcomes. All statistical analyses were performed with SAS version 9.1 (SAS institute, Cary, NC).

## 3. Results

The general characteristics and demographics of the patients, including age, gender, cancer stage, CCI score, and incurred medical care cost, are presented in Table 1. Patients under the age of 49 accounted for over 50% of breast cancer patients, while the highest proportion of colon (31.6%), stomach (33.7%), and lung (43.0%) cancer patients were aged 60–69 years. Although colon cancer did not differ significantly between genders, men made up ≥70% of stomach and lung cancers patients. Because subjects were undergoing surgical treatment, the majority of patients were in the early cancer stages.

The CCI score was calculated based on claims data and varied widely over the four cancer types. The majority of colon cancer patients had a CCI of three or higher (49.6%), while the CCI scores of the stomach cancer patients were distributed relatively evenly.

The association between CCI score and the medical care cost is presented in Table 2. The medical care cost is the highest in CCI = 3 group at colon, stomach, and lung cancer patients. But, in breast cancer, the CCI score is indifferent variable.

The effect of CCI calculated based on both medical records and costs is presented in Table 3. According to these results, a higher CCI number increased the medical care cost of stomach and lung cancer patients 1.05- and 1.06-fold, respectively, but did not significantly increase breast or colon cancer costs.

Comorbidities had varying effects on medical care cost depending on the cancer types with which they presented. The breast cancer patients with chronic obstructive pulmonary disease (COPD) had 1.44-fold higher medical costs than did breast cancer patients without COPD, and colon cancer patients with cerebrovascular disease (CVD) incurred 1.24-fold higher medical costs than did patients with the same cancer type but no CVD. For patients with lung cancer, those with COPD had 0.79-fold lower medical care costs compared to patients without COPD.

Among the breast cancer patients with COPD, a lower CCI increased medical cost by approximately 1.23-fold, in which the medical care costs of the CCI = 0 group were 1.23-fold higher than those of the CCI = 1 group. For patients with colon cancer, the CCI = 1 group with diabetes mellitus (DM) incurred 1.35-fold higher medical care costs than did the CCI = 0 group; in fact, the CCI = 1 group with DM was the group with the highest medical care costs, spending more than any of the other CCI groups with colon cancer. Patients with lung cancer in the CCI = 3 group with COPD as one of the comorbidities had lower medical care costs than other lung cancer patients; for example, they had a 0.74-fold lower cost than the CCI = 0 group.

In the meantime, the CCI calculated using claims data was associated with significantly reduced colon cancer medical care costs (odds ratio: 0.85), with a significance level of 95%. Although myocardial infarction (MI) and DM were the comorbidities most closely associated with increased medical care cost for breast cancer, there was no comorbidity significantly correlated with other cancer types. At this time, MI reduced medical care costs by approximately 70%, and DM was associated with a 40% cost increase.

When the effect of comorbidity on medical care costs was examined by CCI, the breast cancer patients in the CCI = 1 group with COPD had 3.38-fold higher medical care costs than did the CCI = 0 group with the same cancer. Colon cancer patients with DM in the CCI = 2 group paid approximately 14% higher medical care costs than the relevant CCI = 0 group. Lung cancer correlated with COPD, since the CCI = 3 group with COPD incurred 2.11-fold higher medical care costs compared to the CCI = 0 group. Stomach cancer, on the other hand, did not have any significant association.

Cancers and comorbidities were clustered according to fatality and chronic or acute conditions, respectively. Cases of breast or colon cancer with a chronic comorbidity (such as DM and COPD) incurred higher medical care costs than did patients with the same cancers and acute comorbidities (such as CVD and MI), even after adjusting for control variables (Table 4). However, acute comorbidities significantly decreased medical care costs for patients with stomach and lung cancers, compared to patients with these cancers and chronic comorbidities.

## 4. Discussion

The results of this study are consistent with previous reports that CCI or comorbidities are the driving force behind medical care costs. In particular, lung cancer had the highest CCI-related medical care cost, followed by stomach cancer [19].

Previous studies have reported that higher CCI in patients with renal failure or head/neck cancer had increased medical costs and that CCI = 2 patients' medical costs had the highest rate of increase [20, 21]. Considering individual comorbidities in this study, breast cancer patients with COPD spent more on treatment than did patients without COPD; in general, a lower CCI score was associated with relatively decreased medical care costs. Therefore, not only was CCI an important indicator in the economic burden of cancer, but each type of comorbidity was also a factor. These trends, in particular, were found among colon and lung cancer patients. Colon cancer patients with DM in the CCI = 1 group spent more on medical treatment than any other group, except for lung cancer patients with COPD and a CCI of 2 or higher.

In addition, the results of this study regarding the protective or positive effect of comorbidities are consistent with previous reports that frequent hospital visits led to early cancer detection. Higher CCI scores were associated with decreased medical costs among breast cancer patients with DM, though nonsignificant, and in lung cancer patients with COPD. Because breast cancer is primarily treated surgically, the existence of comorbid diseases is not a critical factor in determining the need for surgery timing [8]. Therefore, continuous monitoring during frequent hospital visits is helpful in deciding the appropriate timing of surgery, thus reducing the medical care cost [22]. Further, interactions between various medications for comorbidities influence cancer outcomes [18]. This correlation was observed when patients with breast cancer and COPD in the CCI = 0 group were compared with the low-CCI (CCI = 1) group. Although there was a 1.23-fold difference in medical care costs between the two groups, the difference was not significant. In fact, in breast cancer patients, there was a larger gap between the CCI = 1 group and the groups with a higher CCI score, indicating that the CCI = 1 group, with infrequent hospital visits, consistently incurred higher medical care costs. This trend was also observed in colon cancer patients; the colon cancer patients with low CCI scores had higher medical costs than those with high CCI scores associated with DM. This is consistent with previous reports that the administration of drugs to treat comorbidities reduces the risk of colon and breast cancers, causing a positive effect on health outcomes [23–31].

When patients' CCI scores and the medical care cost were examined, higher CCI scores were associated with higher medical care costs for patients with lung and stomach cancers, since we evaluated subjects who underwent surgical treatment (Table 1). For cancers with a range of developed alternative therapies, such as stomach and lung cancers, whether to perform surgical treatment is frequently determined by existing comorbidities, and patients with high CCI scores are treated in a manner as noninvasive as possible [8]. In other words, lung and stomach cancer patients were only treated surgically when a specific comorbidity necessitated a more invasive treatment; the surgeries were riskier and more complex due to the associated comorbidities, and so this could be considered a factor in the increased medical care cost [32].

In conclusion, comorbidity is more than simply a confounding factor and should be considered a predictor of the medical care cost. The type of comorbidity influences the pattern and availability of treatments and therefore has a profound effect on the economic burden. This may be due to the interaction of drugs administered to treat various comorbidities, as well as the potential for early cancer detection with frequent hospital visits. In addition, because the effect of comorbidities varies for each type of cancer, comorbidity types should be considered as a prognostic factor for the medical care cost, rather than a simple CCI score.

Recently, the landscape of cancer treatment is changing dramatically. In particular, computed tomography and magnetic resonance imaging can let detect in the early stage. So many cancer patients can survive 5 more years [33]. In addition, Koreans can get the national cancer screening program regularly. So the medical care cost will be decreased gradually. And the disease preventive cost like health education program cost or health behavior managing cost will be increased rapidly.

## Key Points

We find that comorbidity has to be reevaluated because of its pros and cons effect. For example, higher CCI score decreased the medical care cost by 10% among breast cancer patients with DM. In addition the correlation with each comorbidity and high morbidity rate by types of cancer was found. That is, for the survey on comorbidity measured with the medical records breast cancer was considerably related with DM, colon cancer and lung cancer were closely associated with CVD, and lung cancer showed a close correlation with COPD.

## Figures and Tables

**Table 1:** General characteristics and demographics of patients treated for breast, colon, stomach, and lung cancers over the study period.

Cancer	Breast	Colon	Stomach	Lung
	*N* (%)	*N* (%)	*N* (%)	*N* (%)
Total	287	272	614	391
Age				
≤49 years	145 (50.5)	44 (16.2)	130 (21.2)	46 (11.8)
50–59 years	83 (28.9)	65 (23.9)	148 (24.1)	108 (27.6)
60–69 years	48 (16.7)	86 (31.6)	207 (33.7)	168 (43.0)
≥70 years	11 (3.8)	77 (28.3)	129 (21.0)	69 (17.6)
Sex				
Female	287 (100.0)	120 (44.1)	184 (30.0)	101 (25.8)
Male	0 (0.0)	152 (55.9)	430 (70.0)	290 (74.2)
Stage				
None	2 (0.7)	1 (0.0)	1 (0.2)	0 (0.0)
1	115 (40.1)	47 (17.3)	297 (61.6)	205 (52.5)
2	116 (40.4)	93 (34.3)	69 (14.3)	76 (19.4)
3	51 (17.8)	78 (28.8)	61 (12.7)	110 (28.1)
4	3 (1.0)	53 (19.6)	54 (11.2)	0 (0.0)
CCI score				
0	134 (46.7)	33 (12.1)	123 (20.0)	269 (68.8)
1	76 (26.5)	52 (19.1)	142 (23.1)	86 (22.0)
2	48 (16.7)	52 (19.1)	165 (26.9)	0 (0.0)
3+	29 (10.1)	135 (49.6)	184 (30.0)	36 (9.2)
Average medical care cost (won)	5,143,328	9,568,920	7,941,467	4,346,660
Average length of stay (days)	24.4	22.6	23.6	17.4

**Table 2:** Association the score of CCI and medical care cost based on medical records.

	Breast cancer		Colon cancer		Stomach cancer		Lung cancer	
	*n*	Mean (won)	*n*	Mean (won)	*n*	Mean (won)	*n*	Mean (won)
CCI = 0	134	5,219,469	33	9,200,986	123	7,792,501	269	4,323,556
CCI = 1	76	5,153,355	52	8,181,566	142	7,702,013	86	4,315,535
CCI = 2	48	4,873,152	52	9,465,901	165	7,736,546	0	0
CCI = 3	29	5,212,408	135	10,232,929	184	8,409,604	36	4,593,652

**Table 3:** Association among associated diseases, CCI, and medical care cost based on medical records.

	Breast cancer		Colon cancer		Stomach cancer		Lung cancer	
	*β*	95% CI	*β*	95% CI	*β*	95% CI	*β*	95% CI
Age group	1.08	0.94–1.24	1.01	0.95–1.08	0.95^*∗*^	0.91–0.99	0.95^*∗*^	0.90–1.00
Gender	—	—	1.06	0.98–1.15	1.03	0.98–1.08	0.94	0.84–1.05
Stage	1.06^*∗*^	1.00–1.12	1.10^*∗*^	1.06–1.15	1.09^*∗*^	1.07–1.11	1.09^*∗*^	1.03–1.16

Each comorbidity disease								
MI	0.71	0.34–1.49	2.56	0.55–11.77	0.92	0.47–1.79	0.93	0.53–1.61
CHF	—	—	0.93	0.67–1.29	1.07	0.63–1.81	1.48	0.57–3.87
CVD	1.22	0.72–2.06	1.24^*∗*^	1.00–1.54	1.10	0.71–1.71	0.79^+^	0.61–0.97
COPD	1.44^+^	1.01–2.11	0.85	0.42–1.72	1.36	0.77–2.39	1.01	0.81–1.25
DM	1.04	0.50–2.15	1.06	0.27–4.27	0.91	0.70–1.18	0.98	0.82–1.16

Charlson Comorbidity Index								
CCI	0.93	0.81–1.06	1.01	0.95–1.08	1.05^*∗*^	1.01–1.09	1.06^+^	1.00–1.13

Interaction between CCI and each comorbidity^a^								
MI								
CCI = 1	—	—	—	—	—	—	1.11	0.29–4.15
CCI = 2	—	—	—	—	—	—	1.20	0.69–2.07
CCI = 3	0.71	0.34–1.49	—	—	0.99	0.88–1.11	1.29	0.62–2.69
CHF								
CCI = 1	—	—	—	—	—	—	0.77	0.43–1.36
CCI = 2	—	—	—	—	—	—	0.80	0.57–1.13
CCI = 3	—	—	—	—	0.96	0.86–1.06	0.84	0.48–1.47
CVD								
CCI = 1	—	—	0.77	0.18–3.27	0.69	0.33–1.43	1.06	0.74–1.53
CCI = 2	1.22	0.96–1.56	0.82	0.42–1.61	0.83	0.58–1.20	1.04	0.83–1.31
CCI = 3	—	—	0.88	0.54–1.43	1.01	0.92–1.10	1.02	0.73–1.42
COPD								
CCI = 1	1.23^+^	0.96–1.56	0.88	0.48–1.61	1.07	0.75–1.53	0.99	0.87–1.14
CCI = 2	1.05	0.85–1.29	0.93	0.67–1.29	1.02	0.85–1.22	0.86^*∗*^	0.74–0.99
CCI = 3	0.89	0.65–1.23	0.98	0.78–1.24	0.97	0.92–1.02	0.74^*∗*^	0.59–0.93
DM								
CCI = 1	—	—	1.35^+^	1.01–1.91	1.24	0.89–1.73	1.14	0.70–1.85
CCI = 2	—	—	1.26^*∗*^	1.03–1.54	1.11	0.94–1.31	0.94	0.71–1.24
CCI = 3	1.04	0.50–2.15	1.18^*∗*^	1.00–1.40	0.99	0.94–1.04	0.77	0.54–1.10

^*∗*^ *p* < 0.05.

^+^ *p* < 0.10.

^a^Reference variable: CCI = 0.

MI, myocardial infarction; CHF, congestive heart failure; CVD, cerebrovascular disease; COPD, chronic obstructive pulmonary disease; DM, diabetes mellitus.

**Table 4:** Association among comorbid characteristics and medical care cost based on claim data.

	Breast/colon cancer	Stomach/lung cancer
Chronic disease	Unadjusted: *β* = 1.84^+^ (CI: 1.29–1.57) Adjusted^a^: *β* = 1.79^*∗*^ (CI: 1.64–1.86)	Unadjusted: *β* = 1.03 (CI: 0.93–1.15) Adjusted^a^: *β* = 1.63 (CI: 0.89–2.96)
Acute disease	Unadjusted: *β* = 1.03 (CI: 0.92–1.17) Adjusted^a^: *β* = 1.21^+^ (CI: 1.00–1.49)	Unadjusted: *β* = 0.03 (CI: 0.94–1.17) Adjusted^a^: *β* = 0.85^*∗*^ (CI: 0.74–0.99)

^*∗*^ *p* < 0.05.

^+^ *p* < 0.10.

^a^Control variable = sex, age, and stage of cancer.
